# Hemostatic effect and distribution of new rhThrombin formulations in rats

**DOI:** 10.2478/intox-2014-0032

**Published:** 2015-03-04

**Authors:** Jan Kochan, L'udmila Schmidtová, Irina Sadloňová, Andrej Murányi, Jana Zigová, Marta Múčková

**Affiliations:** 1 hameln rds, Horná 36, Modra, Slovak Republic; 2 Faculty of Pharmacy, Department of Galenic Pharmacy, Comenius University, Bratislava, Slovak Republic

**Keywords:** rhThrombin, hemostatic effect, histopathology, regeneration, peripheral bleeding, biodistribution, plasma absorption, liver injury, rat

## Abstract

Recombinant human thrombin (rhThrombin) is a potential hemostatic alternative to bovine and human plasma-derived thrombin. Hemostatic, liver regeneration effect and plasma concentrations of rhThrombin (SCILL) tested in the form of solution and hydrogels (thermo-sensitive poloxamer gel and carbomer gel; hameln rds) were evaluated.

In the bleeding model, rhThrombin was applied locally on the bleeding site. The time to hemostasis was measured. The rhThrombin in liquid form as well as the thermo-sensitive gel forming formulation significantly reduced the bleeding time in comparison to saline. In the regeneration model, a cut in the form “V” was made on the liver and rhThrombin in both formulations was applied at defined concentrations to the wound for 5 min. The rats survived 1, 3 and 5 days after the injury and treatment. Histological examination showed better results in the group treated with rh Thrombin gel in comparison to the liquid form – solution; differences were insignificant.

Low [^125^I]-rhThrombin radioactivity was evaluated in plasma after topical application (solution and both hydrogels) at hemostatic effective doses to partial hepatectomy in rats. Locally applied rh Thrombin on the rat damaged liver tissue never reached pharmacologically active systemic levels. The plasmatic levels and the content of this active protein in injured liver tissue were lower after application of its hydrogels versus solution.

## Introduction

Thrombin is a pluripotent hemostatic factor, which promotes coagulation, thrombosis, and local vasoconstriction. This makes topical thrombin an ideal agent for the promotion of hemostasis during surgical procedures (Lew & Weaver, 2008; Cheng *et al.*, 2009; Croxtall & Scott, 2009). More than 1 million surgical patients annually are exposed to thrombin products as an adjunct to hemostasis during surgical procedures (Lawson, 2006).

Until recently, most commercially available thrombin products were isolated from bovine or human plasma, but sourcing thrombin from plasma carries the potential risk of transmitting plasma-borne pathogens of *e.g.* HIV, hepatitis B and C, Creutzfeldt-Jakob disease or bovine spongioform encephalopathy (Regan, 2002). The use of bovine thrombin can also lead to immunological reactions and related post-operative complications (Ortel, 2001). Advances in molecular biology in the last decade have led to investigation and approval of the hemostatic alternative – recombinant human thrombin (rhThrombin). Although rhTrombin has a similar efficiency and safety profile as thrombin from bovine or human plasma, it carries a significantly lower risk of immunologic impact (Chapman, 2007) without potential risk of viral or prion transmission.

Commonly existing, marketed thrombin products are mainly in form of solutions or powder for solution with low viscosity (Lew & Weaver, 2008). Their disadvantage is rapid evaporation and flow out from the application site, which requires frequent application. Moreover, application devices like gauzes or sterile sponges have to be often used as carriers during application. Thrombin semi-solid product should be a reasonable improvement, which enables application directly from the container or by gloved surgical hands. The adhesive hydrogels showed strong adhesiveness to soft tissues and mucous layers and demonstrate also hemostatic properties. Higher viscosity and mechanical structure enhance the hemostatic effect and allow application in areas inaccessible to commonly used application devices. New thermo-sensitive poloxamer (Pluronic^®^ F 127) – based *in situ* gel forming formulation and carbomer gel (Carbopol^®^ 980) were tested in order to confirm their efficiency and safety.

## Methods

### Animals

Male and female Wistar rats (300–350 g) were obtained from Velaz Praha. The rats had free access to food and water and were housed under regulated conditions of temperature (22 ± 2 °C), relative humidity (55 ± 5%) and illumination (12h light/darc cycle). All animal procedures were in compliance with the Animal Welfare Act, the Guide for the Care and Use of Laboratory Animals, and the Office of Laboratory Animale Welfare.

The experiments were approved by the State Veterinary and Food Administration of the Slovak Republic, Decision Č.k. Ro-2229/13-221 and according to the Government Order 377/2012.

### Hemostatic effect

In a bleeding model, the anesthetized rats (2.5% isofluran in a gas mixture of 70% N_2_O and 30% O_2_) were pretreated with heparin (100 IU) i.v. 5 min before surgery. The rhThrombin (solution) was administered on the bleeding site in a gauze soaked in 0.15 mL solution of 100, 200, 400, 800 and 1 000 IU of rhTrombin or saline and rhThrombin thermo-sensitive gel (500, 800 and 1 000 IU) was applied directly on the bleeding site. The time to hemostasis (TTH) was measured. The results are expressed as% of TTH decrease in comparison with control. The data were statistically analyzed using Student′s t-test for paired data, evaluated by Statgraphic centurion.

### Regeneration

In the anesthetized rats a cut in the form of “V” was made on the median hepatic lobe and gauze with either saline or rhTrombin in both formulations (solution and thermo-sensitive gel) at concentrations of 500 and 800 IU were applied to the wound for 5 min. The abdominal cavity was closed and the rats survived 1, 3 and 5 days after the injury and treatment. Liver tissue samples were processed by standard formol-paraffin method and stained with hematoxylin & eosin and van Gieson. The extent of tissue damage, inflammation and regeneration processes were evaluated microscopically.

### Distribution

For iodination, lyophilized powder of rhThrombin was used. RhThrombin was iodinated with Na^125^I (Institute of Isotopes, Budapest, Hungary) IODO-GEN method according to Markwell and Fox (1978) using Pierce pre-coated iodination tubes (Thermo Scientific). The radioactivity of iodide-125 was measured using the automatic gamma counter Wizard 2 (PerkinElmer, USA).

The distribution experiments were conducted to determine systemic uptake of [^125^I]-rhThrombin from its formulation received *in situ* in Wistar male rats. In the hepatic wound model a cut was made on the lobe of the liver. The radiolabeled solution soaked gauze (1 cm^2^) at the dose of 1025 U and 315 µg proteins (∼10 µCi), the hydrogels (thermo-sensitive gel with 16% w/w poloxamer) and 1.5% w/w carbomer at the dose of 500 U and 155 µg proteins (∼5 µCi) were applied to the injury site for 5 min exposure; liver tissue content and the absorption from tissue to systemic circulation were measured up to 24 h (3 animals/time point). Specifically the goals of distribution experiments were to elucidate the pharmacokinetic parameters, which were calculated by the licensed software Kinetica^™^ TermoFisher.

## Results

### Hemostatic effect

**Figure 1:**
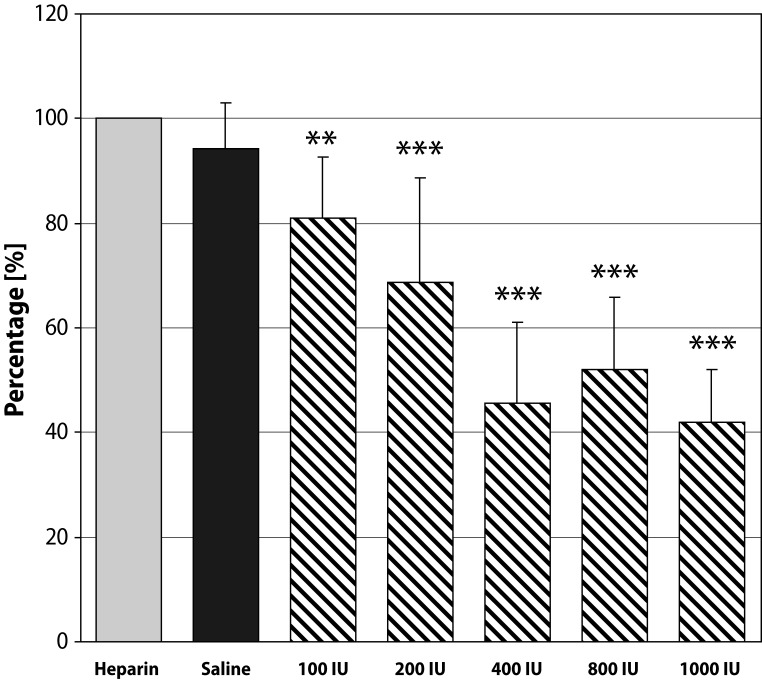
Hemostatic effect of rhThrombin in form of solution. Data expressed as mean ± SD; *p<0.05; **p<0.005; ***p<0.0005.

**Figure 2:**
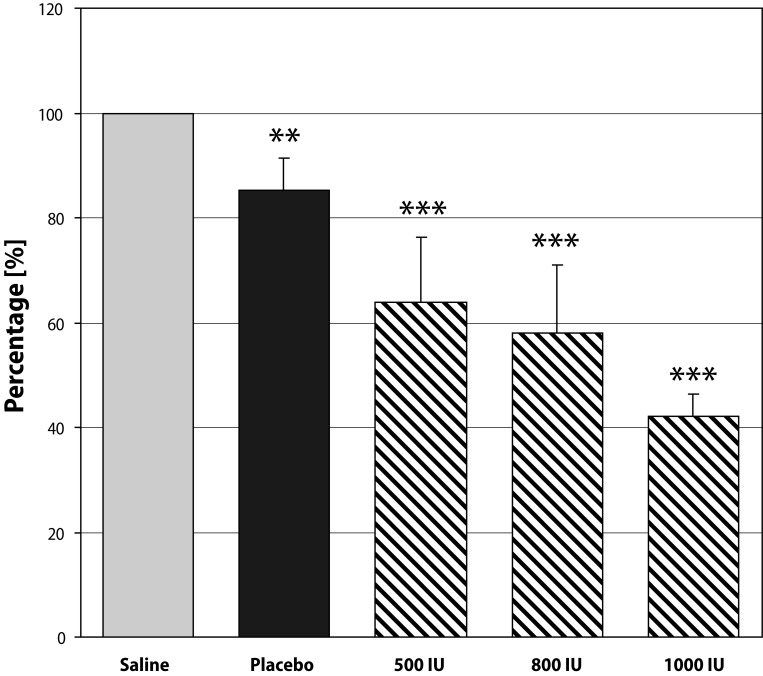
Hemostatic effect of rhThrombin in form of thermo-sensitive poloxamer gel. Data expressed as mean ± SD; *p<0.05; **p<0.005; ***p<0.0005.

In heparin treated Wistar rats TTH values decreased in all concentration of rhTrombin tested, in comparison with controls (saline, placebo). The most pronounced effect was observed after application of rhThrombin at the concentration of 1 000 IU in both formulations used. In this concentration the TTH was shortened to 42.2% in the case of solution and to 42.6% in the case of gel (Figure 1, 2).

### Regeneration

**Figure 3:**
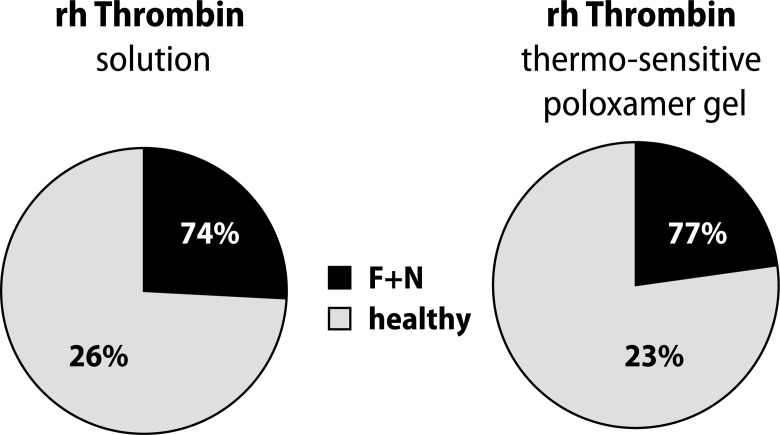
Histopathological results. Difference between liquid and gel form of rhThrombin in liver tissue regeneration 5 days after injury. F+N = fibrosis + necrosis.

The extent of microscopic liver damage and liver regeneration were very different among individual rats treated with either formulation. The mean values indicated a slight improvement in the regeneration of liver tissue in animals treated with rhThrombin in the gel form (Figure 3).

### Biodistribution

**Figure 4:**
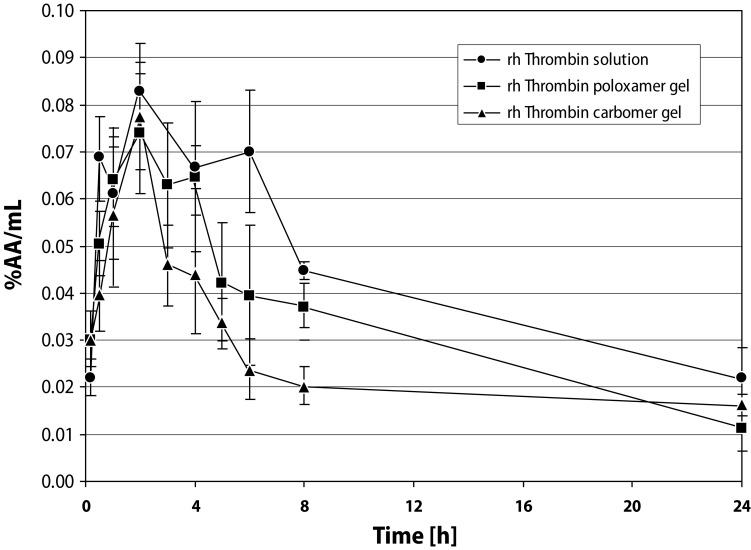
Plasma-concentration profiles of [^125^I]-rhThrombin following application of its formulations in Wistar rat liver wound model. Data expressed as mean ± SD.

The peak of plasma concentration represented only 0.1 and 0.07% of the applied dose of [^125^I]-rhThrombin solution and thermo-sensitive gel, respectively (Figure 4). Following application of [^125^I]-rhThrombin 1.5% carbopol hydrogel, low radioactivity (max. 0.08% AA/mL) was detected in plasma samples collected from 20 min to 24 h post-application. C_max_s were achieved at T_max_ of 2 h. Exposures (AUC_0–24h_) in plasma for rhThrombin solution and lower for poloxamer and carbomer gels were in the order 1>0.8>0.6% AA.h/mL. Terminal elimination half-lives for [^125^I]-rhThrombin were about 10–12 h (longer for carbomer gel approximately 40 h) with mean residence times MRT_0–24_ = 9–10 h (Table 1).

**Table 1:** Basic pharmacokinetic parameters following [^125^I]-rhThrombin formulation application in Wistar rat liver wound model.

Parameter	[^125^I]-rhThrombin formulation		
	Solution	Poloxamr gel	Carbomer gel
C_max_ (%AA/ml)	0.083	0.074	0.078
T_max_ (h)	2	2	2
AUC_0-24_ (%AA.h/ml)	1.01	0.77	0.62
AUMC_0-24_ (%AA.h^2^/ml)	9.7	6.7	5.8
T_1/2_ (h)	12.0	10.2	38.5
k_el_ (1/h)	0.058	0.068	0.018
MRT_0-24_ (h)	9.5	8.8	9.3

The plasmatic levels were lower than thrombin generated in the liver, which represented hepatic tissue/plasma (T/P) ratio (Table 2). The [^125^I]-Thrombin radioactivity in the liver was localized around the site of injury. Very little radioactivity was found at a distant site of the same liver lobe (data not shown).

**Table 2:** Liver tissue to plasma (T/P) content ratio in Wistar rat liver wound model.

Time (h)/ [^125^I]-rhThrombin formulation	0.33	0.67	1	2	4	6	8	24
Solution	28.8±9.4	10.3±3.8	13.5±1.3	14.8±0.6	8.3±3.2	8.0±1.0	4.8±1.0	5.7±1.5
Poloxamer gel	–	–	4.7±1.6	–	2.2±1.8	4.3±2.2	3.4±1.7	7.9±3.4
Carbomer gel	–	–	1.2±0.6	–	1.3±0.5	2.8±1.2	1.9±0.3	1.8±0.2

Data expressed as mean ± SD

## Discussion

Recombinant human thrombin is being developed as an alternative to thrombin products purified from pooled human or bovine plasma, which are currently marketed for topical hemostasis. The first recombinant product on the market developed as an alternative to bovine-derived and human plasma-derived thrombin products was RECOTHROM^®^ (ZymoGenetics).

In our study we investigated the new formulations prepared with the use of a new rhTrombin produced by *E.coli* (Scill) for their hemostatic, liver regeneration and biodistribution effect in rats. Topical rhThrombin was tested in form of a solution and of hydrogels (thermo-sensitive poloxamer gel and carbomer gel; hameln rds).

In the hemostatic model, rhThrombin was applied locally on the injured v. jugularis. The rh Thrombin at a concentration of 1 000 IU reliably/significantly shortened TTH and resulted in more durable hemostasis in the presence of heparin anticoagulation when compared with rhTrombin at lower concentrations. The hemostatic effect of a new rhThrombin formulation (thermo-sensitive poloxamer gel) was comparable with the effect of rhThrombin in form of solution. For clinical practice, a gel formulation is more advantageous because of its strong adhesiveness to soft tissues and mucous layers. Moreover, *in situ* gel formulation can be easily applied in a liquid form, which enables simple manipulation and dosing and direct application on bleeding areas. Its viscosity and mechanical structure are rapidly changed almost immediately after contact with the body surface, thus creating a film on the wound site with semisolid structure to enhance hemostatic effects.

The plasma absorption of rhThrombin from the liver application sites was found to be very low. *In situ* application of rhThrombin solution and mainly the hydrogels poloxamer and carbomer tested relate to local effect, higher in hepatic tissue binding versus active plasma uptake. Generally, locally applied thrombin did not circulate in the blood as free, active molecule as it was rapidly inactivated (<5 minutes) after formation of complexes with endogenous inhibitors (e.g., antithrombin III); these complexes were subsequently cleared by the liver (Buehler 2007, RECOTHROM 2008, EMEA guideline 2009).

## Conclusion

RhThrombin in liquid form significantly shortened the time to hemostasis in comparison with control (saline). A dose-dependent effect was confirmed. A similar effect was determined when rh Thrombin was administered in thermo-sensitive gel-forming formulation.

Histological examination showed better results in the group treated with rhThrombin thermo-sensitive gel in comparison to the solution form, yet the differences were insignificant.

The distribution results show that [^125^I]-rhThrombin in either of its new formulations is but inappreciably absorbed systemically when administered directly to a rat hepatic wound. The absorption of this biologically active protein was found to result in lower plasmatic levels and content in liver tissue on using [^125^I]-rhThrombin hydrogels: thermo-sensitive poloxamer gel and carbomer gel compared to its solution, which manifested hepatic tissue versus plasma content ratios.

A risk assessment for potential systemic toxic effect was conducted and it was concluded that rh Thrombin acts locally, enters the liver tissue negligibly, mainly when applied in the form of hydrogels, and reaches the blood stream to no great extent.
